# Incentive policy optimization of scientific and technological talents and low-carbon economy analysis from the perspective of public health

**DOI:** 10.3389/fpubh.2023.1152346

**Published:** 2023-03-21

**Authors:** Xiaoxuan Yu, Baogui Du

**Affiliations:** ^1^School of Humanity and Law, Northeastern University, Shenyang, China; ^2^Data 61, Commonwealth Scientific and Industrial Research Organisation, Perth, WA, Australia

**Keywords:** public health, talent incentive, low-carbon economy, CO_2_ emission influence, industrial influence

## Abstract

In the face of multiple challenges in stabilizing economic growth, improving people's living quality, and limiting the total amount of CO_2_ emissions, firstly, this study analyzes the incentive and optimization policies of scientific and technological (S&T) talents from four aspects: incentive, cultivation, flow, and evaluation. Moreover, practical suggestions are put forward. Secondly, an optimization model of China's low-carbon economy (LCE) is implemented. The Matlab software can be adopted to solve the economic output of each department in the expected year and obtain the overall economic indicators for 2017 and 2022. Finally, the output influence and CO_2_ emission influence of each industry are analyzed. The research results are as follows. (1) From the viewpoint of public health (PH), the countermeasures and suggestions of the S&T talents policy mainly include four parts: building a complete S&T talents policy system, expanding the coverage group of the policy, strictly implementing the policy of evaluating S&T talents, and improving the guarantee mechanism of relevant talents introduction policy. (2) In 2017, the primary industry, agriculture, forestry, animal husbandry, and fishery, accounted for 5.33%; the secondary industry, the energy sector accounted for 72.04%, and the tertiary industry (service industry) accounted for 22.63%. In 2022, the primary, secondary, and tertiary industry accounted for 6.09%, 68.44%, and 25.47%. (3) From the perspective of the industrial influence coefficient, the coefficient of all sectors is stable during 2017–2022. From the standpoint of CO_2_ emission, China's total CO_2_ emission shows rapidly increasing trend during the same period. This study has vital practical significance and theoretical value for realizing the sustainable development (SH) and transformation of the LCE.

## 1. Introduction

No matter what kind of ideology, development is the eternal theme of human society, it is of great significance to study how to optimize the policy of scientific and technological (S&T) talents, guide relevant skills, explore the development direction and model of S&T skills policy from the angle of public health (PH), and create an excellent social development environment for the talents and guarantee the implementation of the strategy of rural revitalization.

In China's development, energy, as a non-renewable resource, is a key factor in the production function and is extremely important in a country's economic growth (1). The so-called low-carbon economy (LCE) strictly controls the consumption of traditional high-CO_2_ emission and high-pollution energy such as coal and oil utilizing introducing new technologies and developing new power under the guidance of the scientific outlook on development and sustainable development (SD). This can curb greenhouse gas (GHG) emissions to a certain extent, make a favorable balance between economic progress, energy consumption, and environmental protection, thus achieving a social form of fast economic growth without sacrificing the environment, and finally realizing the optimization and upgrading of industrial structure and energy structure (2).

Many domestic and international researchers have studied the relevant aspects of talent policy. Combined with the characteristics of talent resource allocation in higher vocational colleges, Cui (3) analyzed the current situation of talent recruitment in China's vocational colleges to better complete the construction of the national “Double High-levels Plan” and improve the ability of these colleges to cultivate more outstanding talents for the society (3). Gao et al. (4) analyzed the problems existing in environmental accounting information exposure (EAIE) of agricultural and animal husbandry enterprises under LCE. They got a clearer understanding of this aspect, which is conducive to fundamentally proposing countermeasures to improve the level of EAIE (4). Xinsheng et al. (5) took China's rural areas as the research object, explored the status quo of the development of rural personnel, discussed the existing problems in the development of rural human resources, and put forward effective measures to revitalize rural talents, which provided a solid theoretical basis for better rural development (5). Allotey et al. (6) surveyed 10 math and science teachers about their beliefs about talent and their proposed strategies for developing gifted students into skills. The findings suggested that it is necessary to formulate a formal policy on S&T talent education and implement a teacher education plan to solve teachers' beliefs and knowledge about gift and talent education strategy (6). Marco and Lorenzo (7) investigated how promotion incentives affect the productivity of many highly skilled employees by using the three bibliometric thresholds of the national scientific qualifications in the centralized evaluation process of professional development of Italian universities. The results highlighted the importance of promotion incentives as practical motivational tools for public universities and general public organizations (7). From the point of view of theoretical research, foreign scholars have conducted earlier research on S&T talents policy, which mainly starts from education. Chinese scholars pay more attention to the overall grasp of S&T talents policy and advocate establishing the policy system.

Firstly, from the cultivation, evaluation, flow, and incentive of four aspects of S&T talents, the incentive and optimization policy of the skills are studied, and practical suggestions are raised. Secondly, a Chinese LCE optimization model is established to solve the economic output of each industry in the expected year. Finally, the CO_2_ emission influence and output influence of each industry are analyzed. The results reveal that the energy sector is in the middle of the industrial chain and is the pillar industry of the national economy. Still, it is also the primary source of increasing CO_2_ emissions. Some other sectors, such as the equipment manufacturing industry, are located in the lower part of the industrial chain and have a high driving effect on the economy. The innovation of this study lies in the optimization and adjustment of the structure of the industrial sector from the perspective of total carbon emission and total energy consumption control, aiming at the maximization of economic output, the minimization of CO_2_ emission, and the assessment of industrial environmental efficiency, which provides the scientific basis and reference for how to upgrade the industrial structure and how to transition from the traditional economy to the LCE.

## 2. Optimization and incentive of S&T talents policy and low-carbon economy analysis method

### 2.1. Classification of S&T talents incentive policies

**Figure 1:**
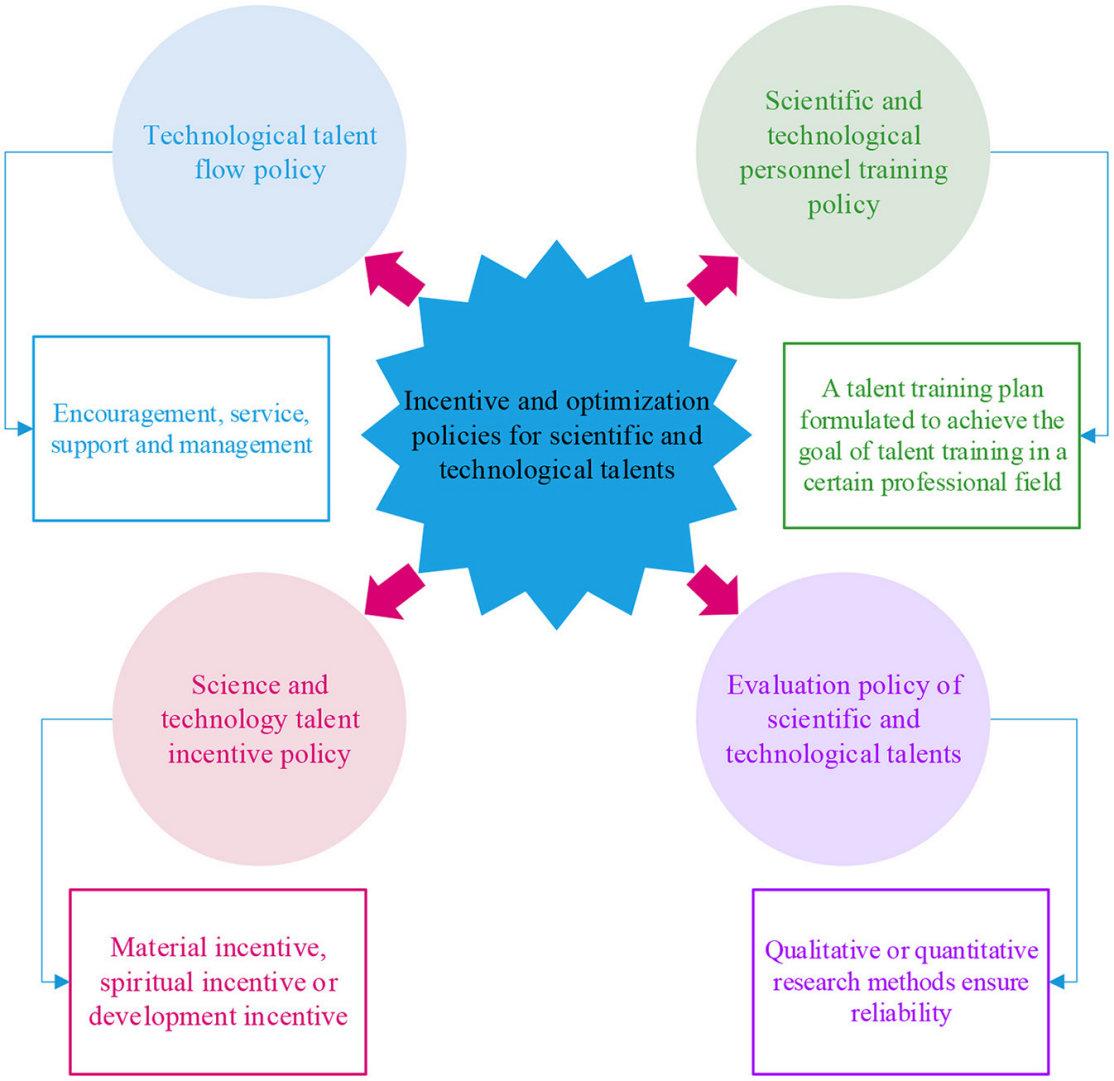
Classification of the S&T talents policy.

S&T talents policy involves a variety of contents, including the incentive, the use, management, and development of S&T talents, etc. In this study, the S&T talents policy is divided into four types, as displayed in Figure 1.

The S&T talents policy can be divided into four types: the flow, cultivation, incentive, and evaluation policy of S&T talents.

#### 2.1.1. The flow policy of S&T talents

The flow of S&T talents mainly covers their introduction and a series of measures to regulate the introduction of relevant skills (8). The introduction policy of S&T talents is aimed at introducing technology personnel, and its principal contents are encouragement, service, support, and management (9). To sum up, the introduction policy of S&T talents refers to the normative criteria for the introduction of relevant skills to meet the needs of local social development under the background of national laws, regulations, and applicable policies to attract and introduce talents (10).

#### 2.1.2. The cultivation policy of S&T talents

As the primary productive force, S&T plays an increasingly prominent role and position in society, and S&T talents, as its primary carrier, have become strategic elements of regional development (11). The relevant policy environment influences the cultivation effect of S&T skills. Scientific talent policy is beneficial to the better development of S&T talents. In summary, the cultivation policy of S&T talents stands for the personnel training program formulated to achieve the talents cultivation goal in a specific professional field. The S&T talent cultivation goal is achieved by standardizing talent cultivation behavior (12).

#### 2.1.3. The incentive policy of S&T talents

It refers to the policy promoting S&T talents' working enthusiasm through spiritual, material, or development incentives (13). The spiritual incentive policy of S&T talents is to inspire these talents by maintaining the skills emotionally and recognizing their achievements. By providing convenience and assistance to S&T talents and their families to solve the concerns of these talents in addition to work, the motivation of such skills can be realized (14). The formulation of the S&T talents incentive policy should not only satisfy the demands of the talents maternally but also pay more attention to their spiritual and future development demands (15).

#### 2.1.4. The evaluation policy of S&T talents

**Figure 2:**
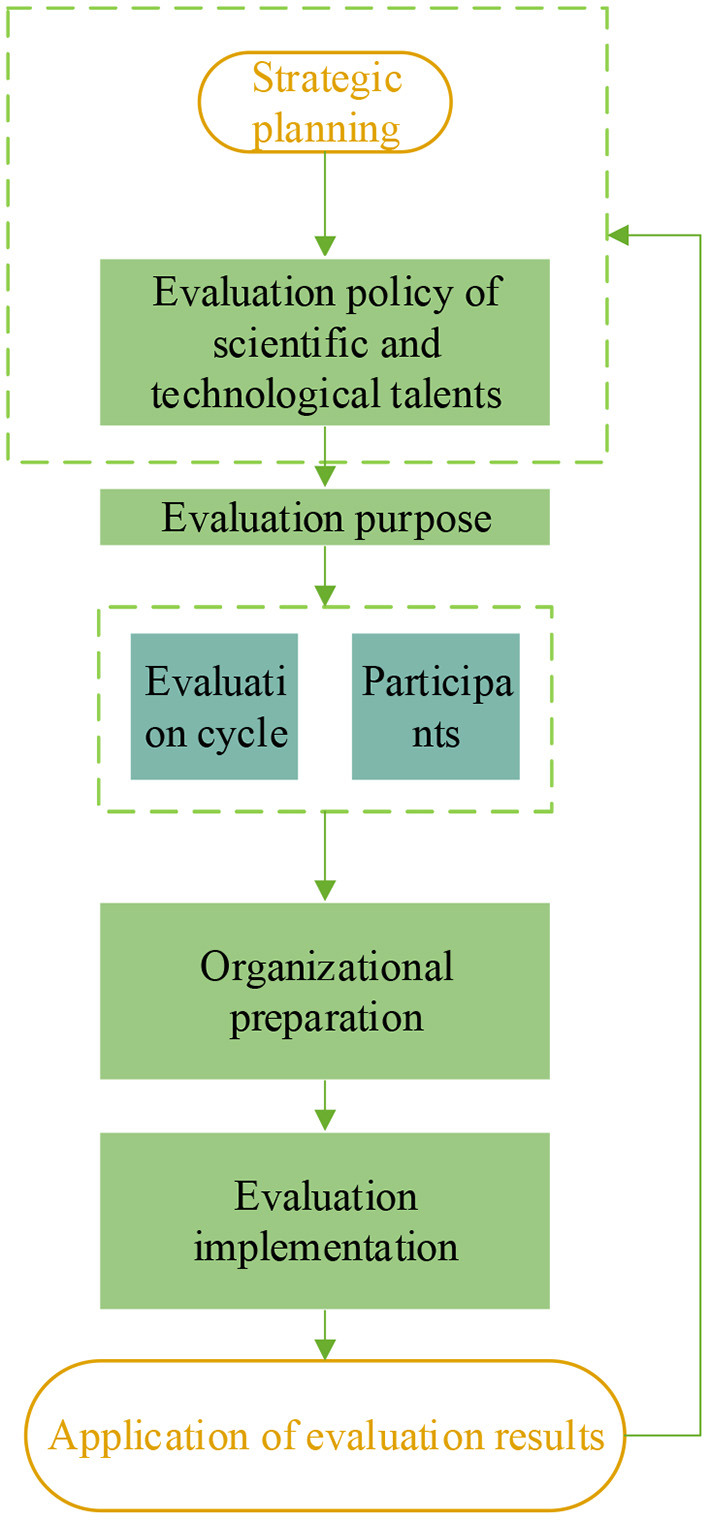
The evaluation mechanism of the evaluation policy of S&T talents.

The evaluation mechanism of the S&T talents evaluation policy is indicated in Figure 2.

**Figure 3:**
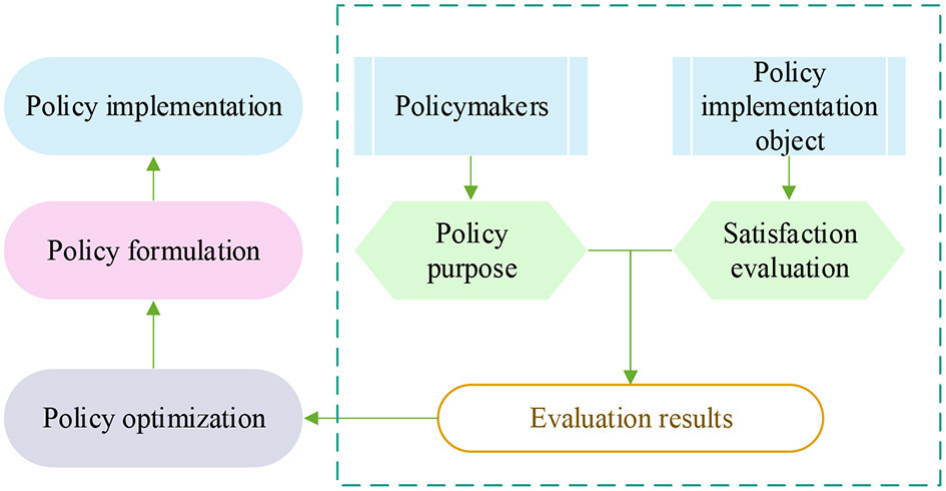
The feedback mechanism of the evaluation policy of S&T talents.

Because a complete cycle can fully reflect the situation of evaluation objects, the evaluation of S&T talents evaluation policy given PH should be carried out in an appropriate and complete cycle (16). The object of evaluation is the provider of evaluation information, which should be composed of members of government, enterprise, and university organizations (17). The evaluation subject should be selected randomly to ensure the objectivity of the evaluation result. The organizational preparation is mainly to prepare for the evaluation of the S&T talents policy (18). The implementation of policy evaluation is to ensure the scientific nature and reliability of the results of the S&T talents evaluation policy from the perspective of PH through qualitative or quantitative research methods so that the evaluation results become an essential basis for optimizing this policy from the point of PH (19). The feedback mechanism of the S&T talents evaluation policy is demonstrated in Figure 3.

From a PH perspective, the evaluation policies of S&T skills would be incomplete without a feedback mechanism. In this perspective, the policy optimization should not only be based on the feedback of the policy evaluation but also on listening to the opinions of the people who implement the policy. Based on the interests of the people, by building a policy information feedback platform, more people can have the opportunity to participate in the process of information feedback on this policy from the viewpoint of PH, to realize the innovation of the existing policy feedback mechanism from relevant perspectives.

### 2.2. The establishment of the low-carbon economy optimization model

The economic output is taken as the decision variable of the LCE optimization model, and the maximum economic output is chosen as the target variable of this model. CO_2_ emission, energy consumption, and economic output structures are selected as constraint conditions to solve the optimization model (20). In this section, the economic output of various sectors in China is taken as the decision variable of the optimization model, and the planning model is constructed to maximize the total economic output. Here, this study puts the control of carbon emission into the constraint to ensure the convenience of solving the model. Then, with energy consumption structure, CO_2_ emission structure, and economic output structure as constraints, the general linear programming method is used to solve the optimization model. The solution method is the Simplex method and interior method. The model constructs the internal connection among economic growth, energy consumption, and CO_2_ emission in an intuitive way. By adjusting the economic output of 41 national economy departments, the model finds the optimal output scheme in China based on controlling the total energy consumption and total carbon emission.

#### 2.2.1. The setting of the objective function

The expression of the objective function of the optimization problem is as follows:


(1) $$\begin{array}{l} \max Z=i^{T}x=x_{1}+x_{2}+\ldots +x_{41} \end{array}$$

*x* refers to the decision variable; *x*_*j*_ stands for the total output of department *j* according to the input–output table, *j* = 1, 2, ….41; *i* signifies a scalar whose coefficients are all *1*, *i* = [1, 1, …, 1]^*T*^. The objective function *Z* is to all of China's output of 41 industries for aggregation, representing the maximization of the total output (21). The number and names of industries are exhibited in Table 1.

**Table 1:** The number and names of industries.

**Number**	**Department name**	**Number**	**Department name**	**Number**	**Department name**
1	Agriculture, forestry, animal husbandry, and fishery	15	Metal products industry	29	Wholesale and retail
2	Coal mining and washing industry	16	General and special equipment manufacturing	30	Accommodation and catering industry
3	Oil and gas extraction industry	17	Transportation equipment manufacturing	31	Financial industry
4	Metal mining and dressing industry	18	Electrical machinery and equipment manufacturing	32	Real estate industry
5	Non-metallic and other mineral mining and dressing industry	19	Communication equipment, computer, and other electronic equipment manufacturing industry	33	Leasing and business services
6	Food manufacturing and tobacco processing industry	20	Instruments and apparatuses	34	Research and experimental development industry
7	Textile industry	21	Crafts and other manufacturing industries (including waste)	35	Comprehensive technical service industry
8	Textile, clothing, shoes, hats, leather, down, and its products	22	Power and heat production and supply industry	36	Water conservancy, environment, and public facilities management
9	Wood processing and furniture manufacturing	23	Gas production and supply industry	37	Resident services and other services
10	Paper printing and cultural, educational, and sports goods manufacturing	24	Water production and supply industry	38	Education
11	Petroleum processing, coking, and nuclear fuel processing industry	25	Construction industry	39	Health, social security, and social welfare industry
12	Chemical industry	26	Transportation and warehousing	40	Culture, sports, and entertainment
13	Non-metallic mineral products industry	27	Postal industry	41	Public management and social organizations
14	Metal smelting and rolling processing industry	28	Information transmission, computer services and software		

Table 1 details the 41 sectors, including agriculture, forestry, animal husbandry and fishery, coal mining and washing industry, oil and gas mining, metal mining and non-metal mining, etc.

The calculation of carbon emissions mainly uses Equation (2):


(2) $$\begin{array}{l} E=AD*EF \end{array}$$

**Figure 4:**
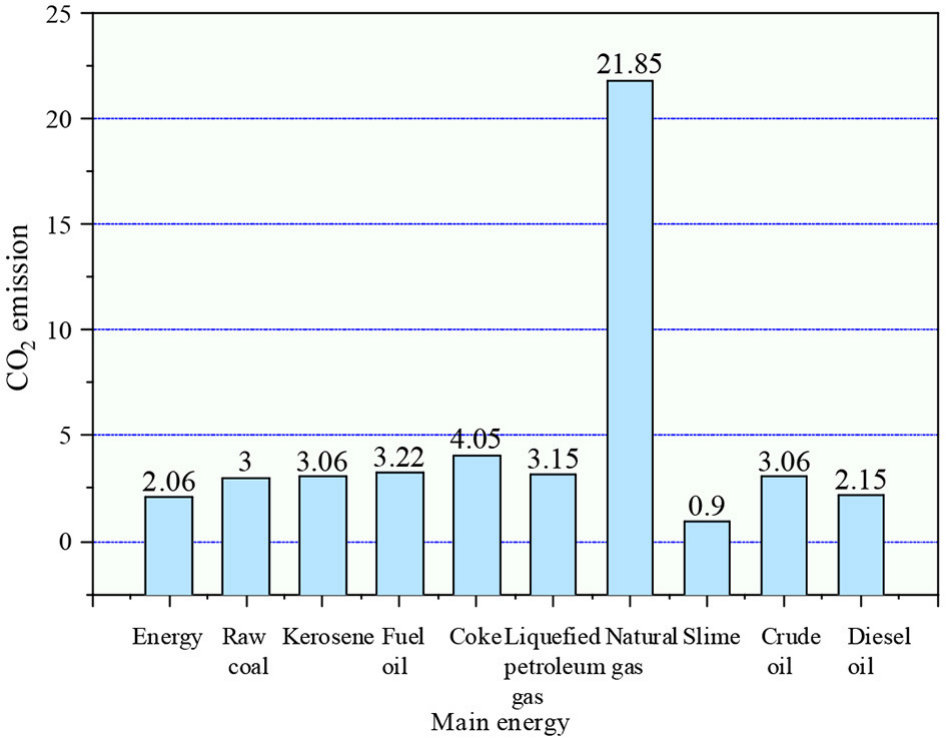
The emission coefficient of the primary energy CO_2_.

*AD* refers to the consumption of fossil fuels, the use of raw materials, and the electricity purchased or exported in the production process during the accounting period. The unit of gas fuel is 10,000 cubic meters (104 m^3^, standard state), and the unit of solid or liquid fuel is tons (*t*). *EF* stands for the CO_2_ factor, namely the CO_2_ coefficient. The emission coefficient of each energy is calculated by the Intergovernmental Panel on Climate Change (IPCC) according to the calorific value of energy combustion, as denoted in Figure 4 (22).

Natural gas is measured in tons of CO_2_/billion m^3^. Everything else is tons of CO_2_/million tons. In Figure 4, the emission coefficient of natural gas is 218,500 tons of CO_2_/billion m^3^, that of raw coal and gasoline is 20,600 tons of CO_2_/million tons, and 30,000 tons of CO_2_/million tons. The CO_2_ emission coefficient of these significant energy sources can be used to calculate the CO_2_ emission influence of each industry.

#### 2.2.2. Setting of constraint condition

**Figure 5:**
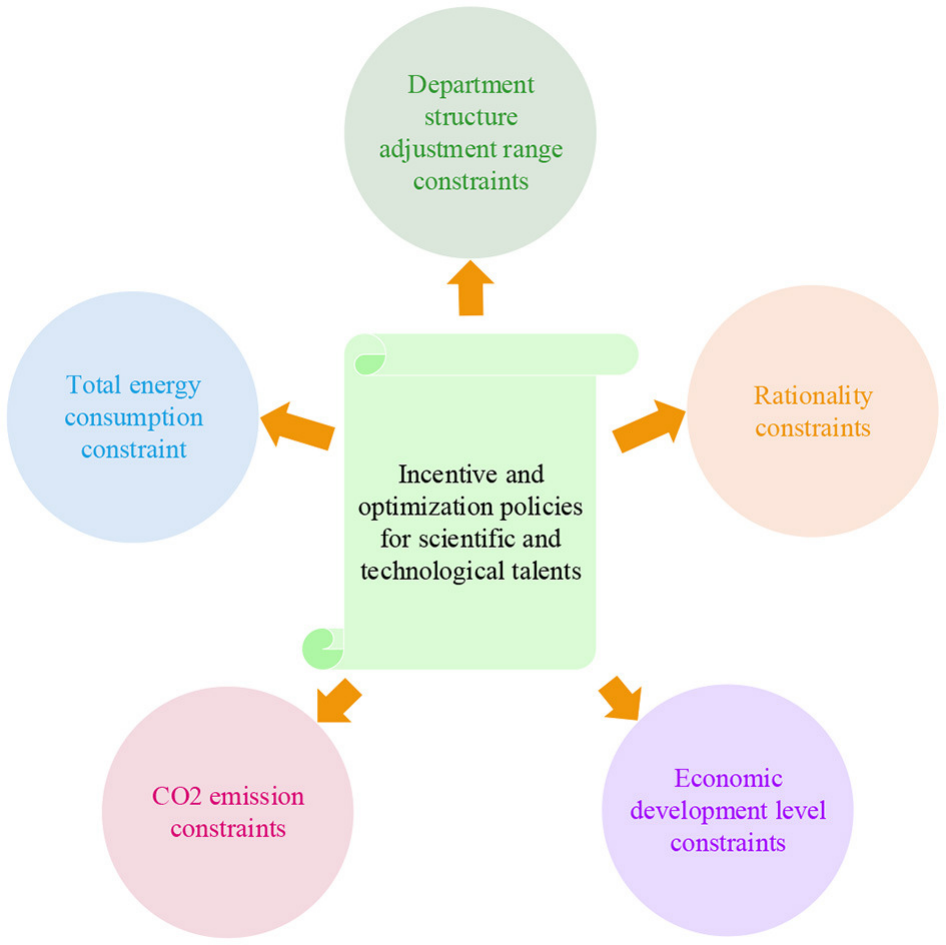
Setting of constraint condition.

The set classification of constraint conditions is revealed in Figure 5.

##### 2.2.2.1. Total energy consumption constraint

This constraint represents the sum of all energy consumed by production activities in all departments, which does not exceed the established predicted value (23). It is expressed by Equation (2):


(3) $$\begin{array}{l} i^{T}A_{e}x\leq e_{f} \end{array}$$

$A_{e}=diag\left(\beta^{T}E\right)$. *E* means the department's direct energy consumption coefficient matrix; β refers to the conversion coefficient of each energy into standard coal, as outlined in Table 2. β^*T*^*E* represents the total specific energy consumption produced by each department, namely, the comprehensive energy productivity, which is 1 × 41 matrix; *e*_*f*_ stands for the predicted energy expenditure of China in 2023 (24). Table 2 presents the energy conversion coefficient.

**Table 2:** Conversion coefficient of energy.

**Name**	**Conversion factor**
Coal	0.7144
Coke	0.9715
Crude oil	1.4288
Gasoline	1.4711
Kerosene	1.4714
Diesel oil	1.4570
Fuel oil	1.4285
Natural gas	13.23
Power	1.230

##### 2.2.2.2. CO_2_ emission constraint

The constraint refers to the sum of CO_2_ emissions from production activities of all sectors, which does not exceed the established predicted value (25), as defined in Equation (3):


(4) $$\begin{array}{l} i^{T}A_{c}x\leq c_{f} \end{array}$$

$A_{c}=diag\left(i^{T}C\right)$. *C* indicates the direct CO_2_ matrix; *i*^*T*^*C* represents the total amount of CO_2_ consumed by each department's production unit output, which is also a 1 × 41 matrix (26). *c*_*f*_ signifies the predicted total CO_2_ emissions of China in 2023.

##### 2.2.2.3. Economic development level constraints

This constraint expresses the sum of the added value of all sectors so that the total amount is not lower than the set forecast of Gross Domestic Product (GDP) (27). The economic development level constraint is written as Equation (4):


(5) $$\begin{array}{l} i^{T}\left(I-A\right)x\geq y_{f} \end{array}$$

*A* means the coefficient matrix of the intermediate consumption of the department; *I* is the identity matrix of 41 × 41; *I*−*A* displays the value-added coefficient matrix; *y*_*f*_ denotes the predicted total GDP of China in 2023.

##### 2.2.2.4. Constraints on the scope of departmental restructuring

It can be represented in Equation (5):


(6) $$\begin{array}{l} lb_{j}\leq x_{f}\leq ub_{j},j=R^{41} \end{array}$$

*lb*_*j*_ and *ub*_*j*_ express the lower and upper bound of the output of department *j* (28). In addition, another purpose of adding this constraint is to eliminate the extreme conditions in the feasible solution set.

##### 2.2.2.5. Rationality constraint


(7) $$\begin{array}{l} x_{j}\geq 0,j=1,2,\ldots ,41 \end{array}$$

*x*_*j*_≥0 means that the output of all departments must be non-negative.

#### 2.2.3. Stationarity test

Generally, the method to test the stationarity of sequence is the Unit root test. In this study, the traditional Augmented Dickey-Fuller (ADF) test and Kommunisticheskaya Partiya Sovetskovo Soyuza (KPSS) test are principally used to ensure the accuracy of the results (29).

##### 2.2.3.1. ADF test

ADF test is based on the Dickey and Fuller (DF) test to expand, its core problem is to test whether the slope coefficient ρ in Equation (7) is 1.


(8) $$\begin{array}{l} Dx_{t}=\alpha +\rho x_{t-1}+\varepsilon_{t} \end{array}$$

*D* represents the difference factor; {ε_*t*_} is a white noise sequence with a mean value of 0, a variance of σ^2^, and an Identical Independent Distribution.

The original hypothesis H0 of the ADF test is that the sequence {*x*_*t*_} has a unit root, that is ρ = 1, that is, {*x*_*t*_} is a non-stationary sequence (30). It is assumed that the value of the characteristic root *P* is within the unit circle and the sequence is stable. According to tradition, the statistics constructed are:


(9) $$\begin{array}{l} t{=}^{\left(\hat{\rho }-\rho \right)}{/}_{se\left(\hat{\rho }\right)} \end{array}$$

$\hat{\rho }$ is the estimated value of the parameter ρ obtained by ρ using the Ordinary Least Squares (OLS).

Under the original assumption, the limiting distribution of the statistic *t* still obeys the general Gaussian distribution. Still, the convergence rate of the intercept term and the slope term is different.


(10) $$\begin{array}{l} \left[\begin{array}{c} T^{1/2}\left(\hat{\alpha }-\alpha \right)\\ T^{3/2}\left(\hat{\rho }-1\right) \end{array}\right]\to N\left(\left[\begin{array}{c} 0\\ 0 \end{array}\right],\sigma^{2}{\left[\begin{array}{cc} 1 & \alpha /2\\ \alpha /2 & \alpha^{2}/3 \end{array}\right]}^{-1}\right) \end{array}$$

Under the alternative hypothesis, regardless of ρ = 0 or |ρ| < 1, The limit distribution of the statistic *t* is still standard normal. When the equation contains lagging items of *Dx*_*t*_, Equation (6) needs to be rewritten as:


(11) $$\begin{array}{l} Dx_{t}=\alpha +\rho x_{t-1}+\overset{\rho }{\underset{i=1}{\sum }}\beta_{i}Dx_{t-i}+\varepsilon_{t} \end{array}$$

##### 2.2.3.2. KPSS test

KPSS test is proposed based on the idea of the traditional hypothesis test. In any hypothesis test, there are two kinds of errors: “false rejection” (Class I error) and “false taking” (Class II error). Based on the original assumption of serial stability, Lagrange Multiplier (LM) statistics can be obtained by the general estimation method.


(12) $$\begin{array}{l} LM=\overset{T}{\underset{t=1}{\sum }}S_{t}^{2}/{\hat{\sigma }}_{e}^{2} \end{array}$$

(13) $$\begin{array}{l} S_{t}=\overset{T}{\underset{t=1}{\sum }}e_{t} \end{array}$$

(14) $$\begin{array}{l} {\hat{\sigma }}_{e}^{2}=\overset{T}{\underset{t=1}{\sum }}e_{t}^{2}/T \end{array}$$

*e* is the estimate of the residual term ε_*t*_ in Equation (7). Of course, if there is a sequence correlation in the residual term, that is, the covariance matrix is not an identity matrix, then:


(15) $$\begin{array}{l} {\hat{\sigma }}_{e}^{2}=\overset{T}{\underset{t=1}{\sum }}e_{t}^{2}/T+2\overset{T}{\underset{t=s+1}{\sum }}w\left(.\right)e_{t}e_{t-s}/T \end{array}$$

*w*(.) stands for variable-weight functions, and various kernel functions choose different smooth ways.

ADF and KPSS methods are employed to test the stationarity of the three sequences. The results of the two tests are compared in Table 3. Among them, the Autocorrelation function (ACF) lag order is selected by minimizing the Schwarz Information Criteria (SIC) criterion, and the intercept term is used as an exogenous variable.

**Table 3:** Test results for stationarity.

**Variable name**	**ADF inspection**		**KPSS inspection**	
	**Original sequence**	**After first-order difference**	**Original sequence**	**After first-order difference**
Ln (GDP)	−1.82	−5.52	0.2	0.097
	(0.68)	(0.001)^***^	(0.03)^***^	(0.15)
Ln (Energy)	−1.41	−4.46	3.01	0.1402
	(0.59)	(0.001)^***^	(0.01)^***^	(0.13)
Ln (CE)	−1.35	−4.53	3.0003	0.1355
	(0.608)	(0.001)^***^	(0.01)^***^	(0.16)

^a^The lag order of the ADF test is selected as 3; the KPSS test truncation lag variable is 1.

^b**^and

*** represent the statistically exact number at 5% and 1%, respectively.

In Table 3, ADF inspection considers that the sequence of GDP, energy consumption, and CO_2_ emissions after logarithmic treatment is a non-stationary I(1) process. All three become stationary processes after first-order differences. KPSS inspection considers that no matter which variable, its original sequence after logarithmic is non-stationary, and after first-order difference, the three sequences become stationary. Therefore, all three variables can be considered as I(1) processes.

## 3. Experimental design and performance evaluation

### 3.1. Recommendations for optimizing S&T talents policy from a PH perspective

**Figure 6:**
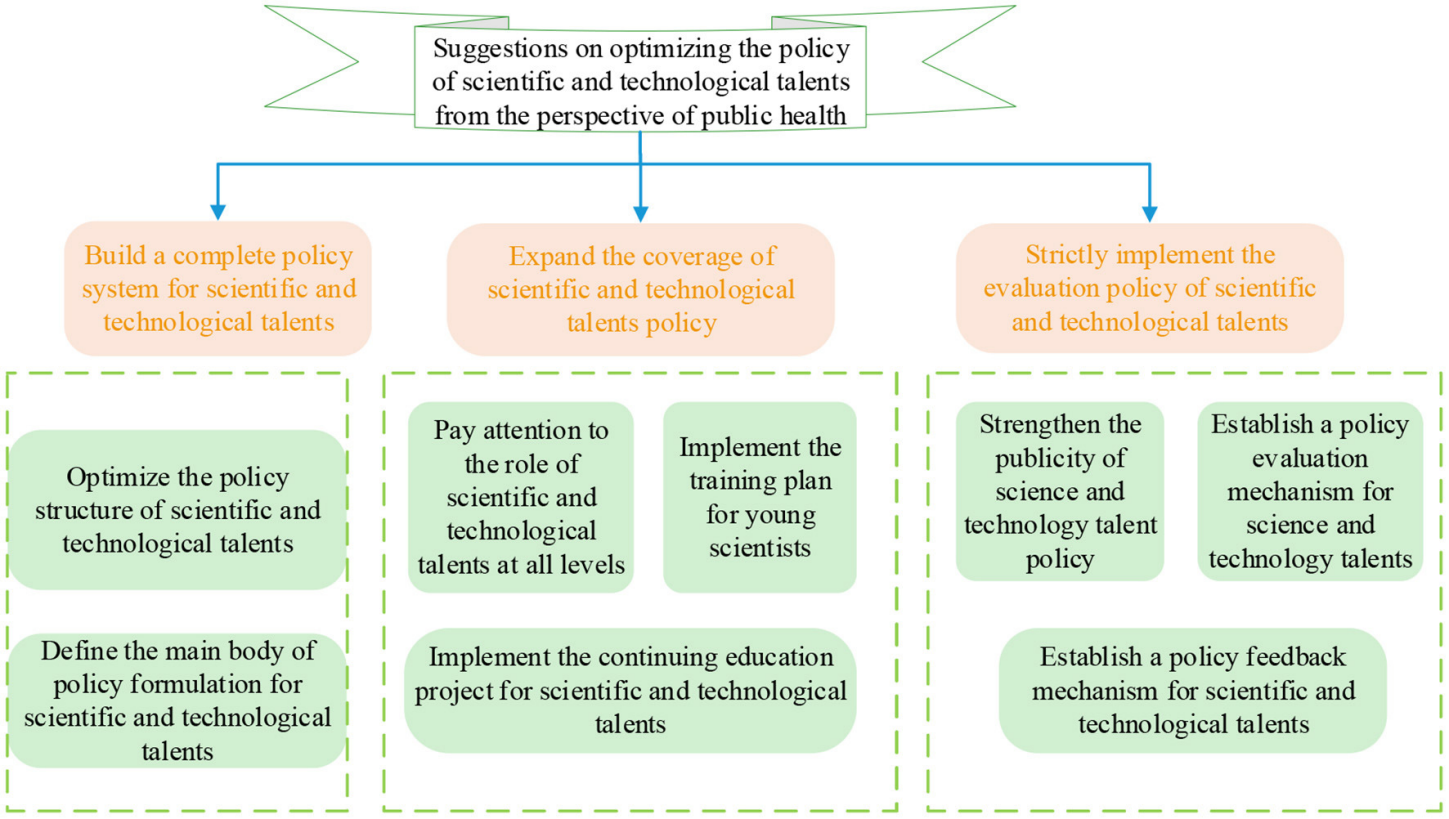
The suggestions for optimizing the S&T talents policy.

In the view of PH, the recommendations for optimizing the S&T talents policy are plotted in Figure 6.

This study puts forward some suggestions on S&T talents policy from the viewpoint of optimizing PH. It mainly covers establishing a complete policy system, expanding policy coverage, strict implementation of such talent evaluation policy, and improving the guarantee mechanism of the talent introduction policy. Establishing a complete policy system includes clarifying the main body of the policy-making and optimizing the structure of this policy. The expansion of policy coverage includes paying attention to the role of such talents at all levels, implementing the training plan for young scientists, and implementing the S&T talents continuing education project. The strict implementation of the S&T talents evaluation policy involves increasing the publicity and establishing the evaluation mechanism and feedback mechanism of the policy.

### 3.2. Solution of low-carbon economy optimization model in China

**Figure 7:**
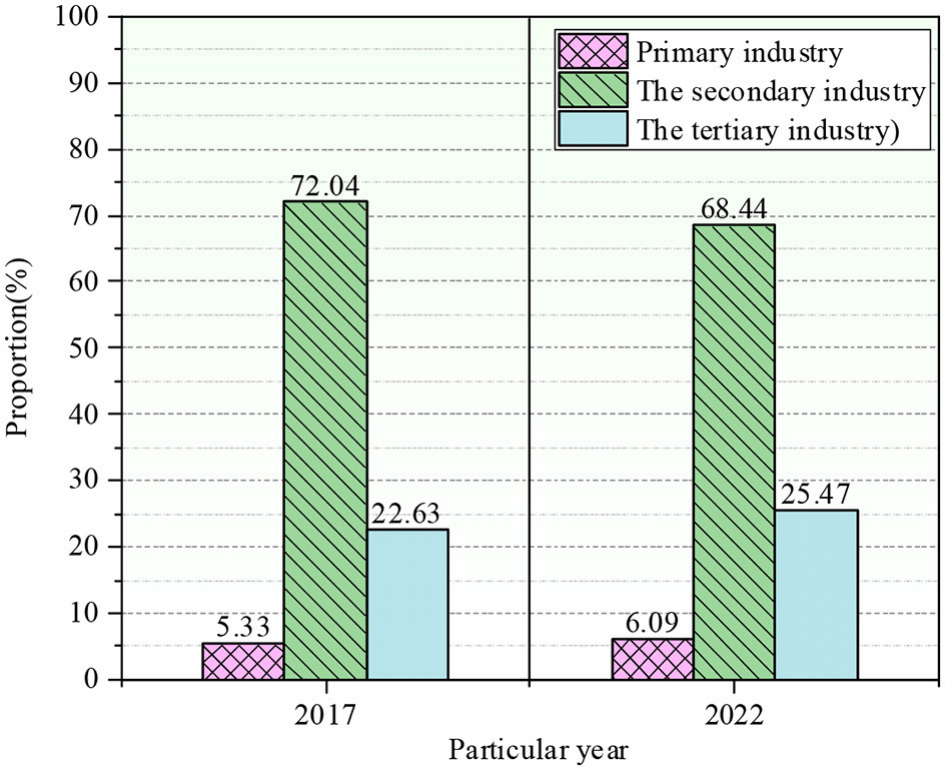
Overall economic indicators for 2017 and 2022.

Matlab has powerful data processing and analysis functions. This study adopts Matlab software to solve the economic output of each department in the expected year, and the overall economic indicators in 2017 and 2022 are obtained, as illustrated in Figure 7.

**Figure 8:**
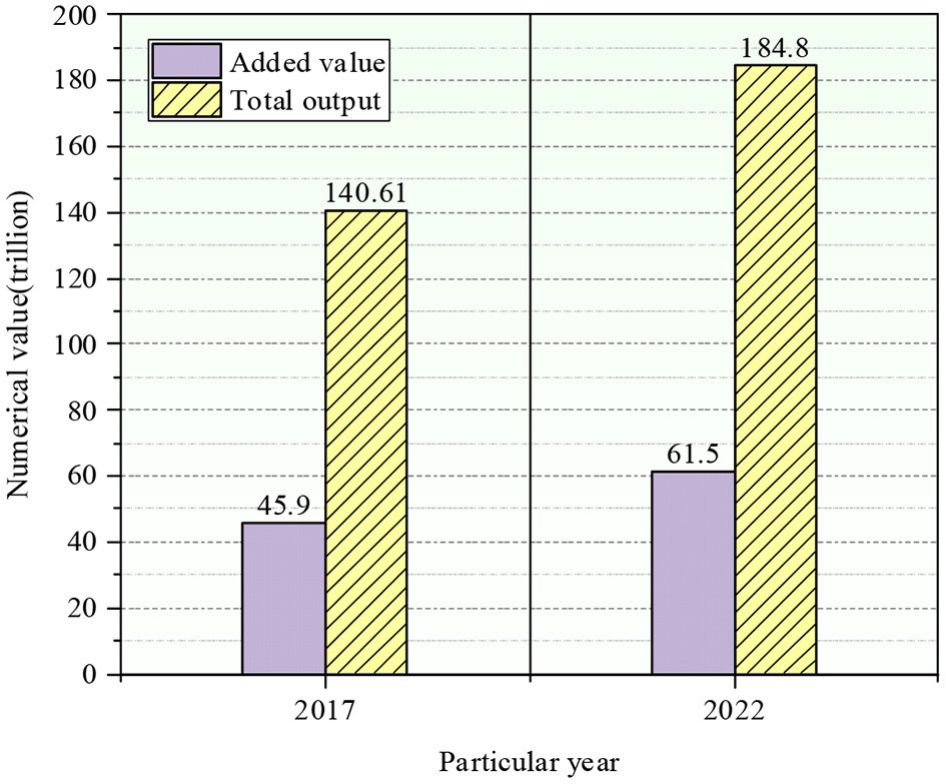
The added value and a total output of the three industries.

In 2017, the proportion of agriculture, forestry, animal husbandry, and fishery in the primary industry was 5.33%, that of energy in the secondary sector was 72.04%, and that of service in the tertiary industry was 22.63%. In 2022, the primary, secondary, and tertiary industries accounted for 6.09%, 68.44%, and 25.47%. The proportion of the primary sector in the overall economy did not change much. The economic added value and the total output of China's three major industries are presented in Figure 8.

Compared with 2017 (base year), the total economic added value of the three industries reached 61.5 trillion yuan, 15.6 trillion yuan more than that of 2017, the total economic output value of the three sectors achieved 184.8 trillion yuan, 44.19 trillion yuan more than that of 2017. The declining trend of the output of the secondary sector is evident, illustrating that the constraints of energy conservation and emission reduction impact the industrial sector as a whole. The proportion of tertiary industry increased slightly.

### 3.3. Analysis of the influence coefficient of various departments

#### 3.3.1. Industrial influence coefficient

**Figure 9:**
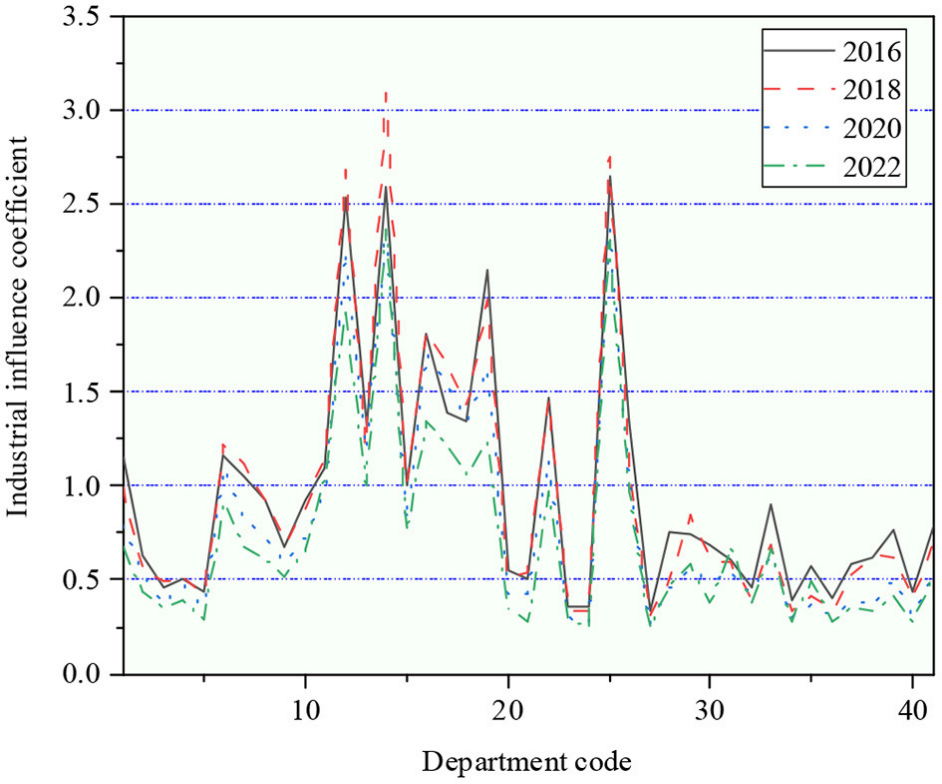
The industrial influence coefficient during 2016–2022.

The industrial influence coefficient from 2016 to 2022 is signified in Figure 9.

The industrial influence coefficient of all sectors in China during 2016–2022 is stable. The influence coefficient of the secondary industry is much higher than that of the primary and tertiary industries as a whole, and most of them are more than the average value of social industries. Thus, it can be found that the secondary sector is still the main driving force of the national economy in China. Among them, the driving effect of agriculture, forestry, animal husbandry, and fishery on China's economy has been declining yearly, with the influence coefficient dropping from 1.15 in 2016 to 0.67 in 2022. In addition to transportation and storage, the industrial influence coefficient of other departments of the tertiary industry is generally lower than the average level of the national economy. Besides, the driving capacity of the transportation industry for the overall economy also presents a downward trend year after year, from 1.35 in 2016 to 0.97 in 2022. It indicates that the service industry plays an insignificant role in driving China's overall economy. In the manufacturing industry, the chemical industry, metal smelting, and calendering industry have the strongest pulling force, with an average influence of more than 2 in 2016–2022. While light manufacturing industries, such as textiles, wood processing, and furniture manufacturing, have a low pull on the overall economy and a downward trend. Among the products and services of the mining industry, the industrial influence of all four sectors was low, maintaining around 0.5 between 2016 and 2022.

#### 3.3.2. The influence coefficient of CO_2_ emission

**Figure 10:**
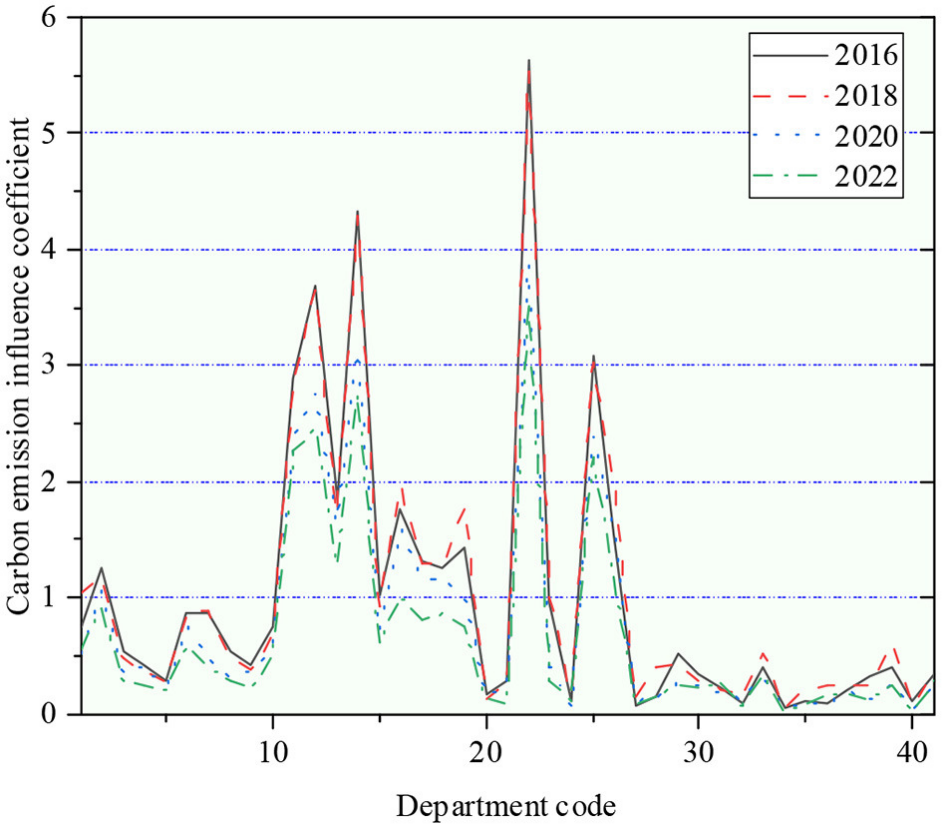
The influence coefficient of CO_2_ emission from 2016 to 2022.

The influence coefficient of CO_2_ emission during 2016–2022 is portrayed in Figure 10.

During 2016–2022, except for a few sectors, the amount of CO_2_ emitted per unit of the added value of all industries in China appeared a downward trend, which means that the carbon productivity of each sector increased with each passing year, illustrating that the “low carbon development” trend of China's economy is generally good. In terms of industrial influence and CO_2_ emission influence, the driving role of the secondary industry is much higher than that of the tertiary and primary industries, and most of them are resource-intensive industries. However, the effect of the tertiary industry on the overall economy has gradually emerged and expanded. In conclusion, the secondary industry will remain a pillar industry in China during the 2016–2022 period. But in terms of trends, the influence of services has risen slightly over this period, while manufacturing has tended to decline. By comprehensively considering the CO_2_ emission and industrial influence coefficients, China should give priority to developing industries with low carbon influence. The scientific development of these sectors with high carbon emissions and high industrial impact has been promoted, mainly through technological improvement to reduce and limit high CO_2_ emissions of these industries.

## 4. Discussion

The first section is the countermeasures and proposals of S&T talents policy from the perspective of optimizing PH. There are four significant countermeasures, namely, the establishment of the S&T talents policy system, the expansion of coverage groups, the implementation of evaluation policies, and the improvement of the introduction of safeguards, each deepens the strategies of two to three minor aspects, respectively. In the second section, by solving the LCE optimization model of China, it is concluded that the proportion of agriculture, forestry, animal husbandry, and fishery in the overall economy has little change. In contrast, the output of the secondary industry has a relatively obvious downward trend, showing that the constraints of energy conservation and emission reduction have an influence on the industrial sector. In the third section, through the analysis of the influence coefficient of various industries, the industrial influence coefficient of all sectors in China during 2016–2022 is stable. Generally, the influence coefficient of the secondary sector is much higher than that of the primary and tertiary industries, and most of them override the average value of social industries. Thereby, it can be drawn that the secondary sector is still the main driving force of the national economy. Liu et al. (31) implemented an energy-saving and emission-reduction decision optimization model from the perspective of a low-carbon supply chain of auto parts (31). Compared with this study, it can be studied from the standpoint of energy conservation and emission reduction and added the perspective of industrial influence.

## 5. Conclusion

The direct purpose of LCE is to reduce emissions of CO_2_ and other GHGs under the premise of ensuring a certain economic growth rate. Its essence is to optimize the industrial structure, improve energy efficiency, and achieve the goal of low-carbon development. China is currently facing multiple challenges such as improving people's quality of life, stabilizing economic growth, and limiting the total amount of CO_2_ emissions. S&T talents incentive policy has vital theoretical value and practical significance for China to realize the transformation of LCE and SD. Firstly, from four aspects of S&T talents evaluation flow, cultivation, and incentive of S&T talents, the study explores incentive and optimization of the S&T talents policy and puts forward effective suggestions. Secondly, a Chinese LCE optimization model is constructed. Using MATLAB software, the economic output of each sector in the expected year can be solved to obtain overall economic indicators for 2017 and 2022. Finally, the output influence of each industry and CO_2_ influence are analyzed. The results of the study are as follows: (1) From the point of view of PH, the suggestions and countermeasures of the S&T talents policy mainly consist of four parts: expanding the coverage group of this policy, establishing a complete policy system, perfecting the safeguard mechanism of such talents introduction policy, and strictly implementing these talents evaluation policy. (2) Compared with 2017, the total economic output of the three industries reached 184.8 trillion yuan, 44.19 trillion yuan higher than that of 2017. (3) Overall, the influence coefficient of the secondary industry is much stronger than that of the primary and tertiary sectors, and the majority exceed the mean value of social industries. Consequently, it can be concluded that the secondary industry is still the main driving force of the national economy in China. The disadvantage is that in the comparable price input–output model used in this study, the price deflator of the tertiary sector is determined by the added value coefficient. Due to data limitations, the added value coefficient in the statistical yearbook cannot cover all sectors. Thus, the added value coefficient of other service industries is used to deflate the remaining industries, which will slightly affect the accuracy of the data. Besides, price factors such as carbon prices and policy trends of various provinces need to be incorporated into the relevant influencing factors in the study content, so as to achieve a more complete development strategy for China's LCE. Therefore, in future studies, the regional development trend of China's LCE will be tracked timely according to the update of data, and the future growth trend will be predicted as far as possible by using rigorous and scientific models based on relevant statistical methods.

## Data availability statement

The raw data supporting the conclusions of this article will be made available by the authors, without undue reservation.

## Ethics statement

The studies involving human participants were reviewed and approved by Northeastern University Ethics Committee. The patients/participants provided their written informed consent to participate in this study. Written informed consent was obtained from the individual(s) for the publication of any potentially identifiable images or data included in this article.

## Author contributions

All authors listed have made a substantial, direct, and intellectual contribution to the work and approved it for publication.
